# PTHF/LATP Composite Polymer Electrolyte for Solid State Batteries

**DOI:** 10.3390/polym16223176

**Published:** 2024-11-14

**Authors:** Elmira Nurgaziyeva, Gulnur Turlybay, Aigul Tugelbayeva, Almagul Mentbayeva, Sandugash Kalybekkyzy

**Affiliations:** 1 National Laboratory Astana, Nazarbayev University, Astana 010000, Kazakhstan; elmira.nurgaziyeva@nu.edu.kz (E.N.); gulnur.turlybay@nu.edu.kz (G.T.); aigul.tugelbayeva@nu.edu.kz (A.T.); 2 Department of Chemical and Materials Engineering, School of Engineering and Digital Sciences, Nazarbayev University, Astana 010000, Kazakhstan; almagul.mentbayeva@nu.edu.kz; 3 Department of Chemistry, School of Sciences and Humanities, Nazarbayev University, Astana 010000, Kazakhstan

**Keywords:** composite polymer electrolytes, LATP, ceramic particles, NASICON-type, lithium-ion batteries

## Abstract

The novel crosslinked composite polymer electrolyte (CPE) was developed and investigated using polytetrahydrofuran (PTHF) and polyethyleneglycol diacrylate (PEGDA), incorporating lithium aluminum titanium phosphate (LATP) particles and lithium bis(trifluoromethanesulfonyl)imide (LiTFSI) salt. Composite polymer electrolytes (CPEs) for solid-state lithium-ion batteries (LIBs) were synthesized by harnessing the synergistic effects of PTHF crosslinking and the addition of LATP ceramics, while systematically varying the film composition and LATP content. CPEs containing 15 wt% LATP (PPL15) demonstrated improved mechanical strength and electrochemical stability, achieving a high conductivity of 1.16 × 10^−5^ S·cm^−1^ at 80 °C, outperforming conventional PEO-based polymer electrolytes. The CPE system effectively addresses safety concerns and mitigates the rapid degradation typically associated with polyether electrolytes. The incorporation of PEGDA not only enhances mechanical stability but also facilitates lithium salt dissociation and ion transport, leading to a uniform microstructure free from agglomerated particles. The temperature-dependent ionic conductivity measurements indicated optimal performance at lower LATP concentrations, highlighting the impact of ceramic particle agglomeration onion transport pathways. These findings contribute to advancing solid-state battery systems toward practical application.

## 1. Introduction

The increasing demand for high-performance energy storage systems has driven significant advancements in battery technology, particularly in the development of lithium-ion batteries (LIBs) [1,2]. These batteries are essential for powering portable electronics, electric vehicles, and large-scale energy storage systems due to their high energy density, long cycle life, and efficiency. However, safety concerns associated with using liquid electrolytes in conventional LIBs—such as flammability, leakage, and instability—have highlighted the need for safer, more stable alternatives [3,4].

Solid-state electrolytes (SSEs) have emerged as promising candidates to address these issues [5,6]. Unlike liquid electrolytes, SSEs offer improved safety, stability, and the ability to suppress dendrite formation. There are a few types of SSEs, including polymer-based and ceramic particle-based systems. Solid polymer electrolytes (SPEs), composed of lithium salts embedded in a polymer matrix, are recognized for their flexibility and enhanced safety in solid-state battery applications. SPEs can effectively suppress lithium dendrite growth by improving interfacial stability with the lithium anode [7]. However, their tendency to crystallize at ambient temperatures leads to reduced ionic conductivity, limiting their practical use [8,9]. On the other hand, inorganic ceramic electrolytes (ISEs) offer high ionic conductivity (10^−3^–10^−2^ S·cm^−1^), excellent electrochemical stability, and strong mechanical properties, but they struggle to maintain effective contact with the electrodes [10].

Among the different types of SSEs, composite polymer electrolytes (CPEs) have gained attention for combining the flexibility of polymers with the high ionic conductivity of ceramic (inorganic) fillers. Various polymer matrices have been studied for use in CPEs, including PEO [11], PVDF [12,13,14], PAN, PVA [15,16], PVP [17], and others. For decades, ion-conductive polymer research has focused on polyethylene oxide (PEO)-based systems [11,18,19]. However, their low ionic conductivity (<10^−6^ S·cm^−1^ at 60 °C), due to crystallinity, limits performance. The low Li^+^ transference number combined with strong EO-Li ion interactions leads to reduced ionic conductivity, as more current is carried by anions rather than Li ions. This lowers the efficiency of lithium transport and increases polarization effects, further degrading the performance of the PEO-based electrolytes. Polytetrahydrofuran (PTHF), with its amorphous structure is emerging as a promising alternative for lithium-ion conduction [20]. PTHF contains fewer oxygen atoms in its polymer backbone than conventional polymer electrolytes, which may help mitigate issues like low ionic conductivity and limited electrochemical stability [21]. Additionally, the terminal groups of PTHF are versatile and can be modified with various functional groups or converted into UV-curable derivatives, increasing its potential for advanced electrolyte formulations [22]. However, the inherent mechanical strength and low melting point of PTHF remain insufficient for practical applications. To overcome these limitations, crosslinking has been explored as an effective strategy to enhance its mechanical properties and create a more robust polymer matrix [20,23]. Although PTHF has been used in the fabrication of solid polymer electrolytes [23,24,25], research on its application in composite polymer electrolytes remains limited. Incorporating inorganic fillers into the PTHF structure is a promising strategy to enhance its performance, forming a composite polymer electrolyte that can be used in practical applications. Currently, extensive research is focused on methods that incorporate various ceramic particles [26,27,28]. NASICON-type fillers, such as Li₁₊ₓAlₓTi₂₋ₓ(PO₄)₃ and Li₁₊ₓAlₓGe₂₋ₓ(PO₄)₃, are well-known for their stability in air and moisture [29]. An additional advantage of these fillers is their ability to accommodate more lithium ions due to the presence of Al³⁺ in their structure, which enhances ionic conductivity [29,30]. Research has successfully demonstrated that composite polymer electrolytes incorporating LLZO and LLZTO nanofillers exhibit high ionic conductivities of 2.6 × 10^−4^ S·cm^−1^ and 5 × 10^−4^ S·cm^−1^ at room temperature, respectively [31,32]. Active fillers enhance Li⁺ transport by incorporating conductive ions into the conduction process. They can create continuous ion channels within the bulk phase to facilitate ion transport. To achieve high ionic conductivity in composite polymer electrolytes (CPEs), careful selection of constituents, compatibility between components (including polymer-polymer and polymer-ceramic interactions), and decisions regarding preparation parameters (such as mass ratios and methods of filler preparation and addition) are crucial. Additionally, the interaction between CPEs and electrodes in lithium-ion batteries (LIBs) plays a significant role [13,33].

In this study, a novel composite polymer electrolyte was designed and fabricated with PTHF polymer and polyethyleneglycol diacrylate (PEGDA) oligomer incorporating LATP ceramic particles and lithium bis(trifluoromethanesulfonyl)imide (LiTFSI) salt. By leveraging the synergistic effects of crosslinking and LATP addition, we seek to create a composite electrolyte that meets the rigorous demands of modern high-performance LIBs. Ionic conductivity for all formulations was tested between 30–80 °C using AC impedance spectroscopy. The highest ionic conductivity value for the PPL15 CPE was 1.57·10^−5^ S·cm^−1^ at 80 °C, surpassing that of conventional PEO-based polymer electrolytes [34]. A significantly lower polarization potential of 30 mV was observed in the lithium plating/stripping cycling tests at current densities of 0.2 mA cm^−2^. The developed CPE system addresses key challenges, such as safety concerns and the rapid degradation of polyether electrolytes, advancing solid-state battery systems toward practical application.

## 2. Materials and Methods

### 2.1. Materials

Hydroxy-terminated poly(tetrahydrofuran) (PTHF, ≥97% purity, Mn~1000), 2-isocyanatoethyl methacrylate (98% purity), poly(ethylene glycol) diacrylate (PEGDA, Mn = 575), dichloromethane (DCM, CH_2_Cl_2_, a water-free, ≥99.8% purity), tetrahydrorofuran (THF, anhydrous, ≥99.9% purity), 2-hydroxy-2-methylpropiophenone (HMPP, ≥97% purity), lithium bis (trifluoromethane) sulfonimide (LiTFSI, 99.95% purity), lithium hydroxide monohydrate (LiNO_3_·H_2_O, ≥98% purity), phosphoric acid (H_3_PO_4_, ≥85% purity), titanium (IV) chloride (TiCl_4_, ≥99.9% purity), ethanol absolute pure (EtOH), aluminum chloride (AlCl_3_, ≥98% purity) were obtained from Sigma-Aldrich (St. Louis, MO, USA). Solvents and chemicals were used without further purification.

### 2.2. Synthesis of LATP Nanoparticles

Analytical-grade LiNO_3_, AlCl_3_, TiCl_4_, and H_3_PO_4_ were used without further purification. The compounds were measured precisely according to the stoichiometric ratio for Li_1.4_A_l0.4_Ti_1.6_(PO_4_)_3_. TiCl_4_ and AlCl_3_ were first dissolved in ethanol (EtOH) and stirred for 30 min. LiNO_3_•3H_2_O and H_3_PO_4_ were then added to the solution with vigorous stirring, continuing for an additional 2 h. The resulting homogeneous sol was poured into Petri dishes and heated at 50 °C to evaporate the solvent, producing a viscous sol-gel. This sol-gel was further aged at 100 °C for 6 h to yield a dry LATP precursor. The dried gel was subsequently pyrolyzed in air at 300 °C for 3 h and then annealed at 800 °C for 2 h.

### 2.3. Synthesis of Methacrylated-PTHF

The modification of hydroxy-terminated poly(tetrahydrofuran) (Mn ≈ 1000) was carried out following reported methods [20]. Initially, 12 g of hydroxyl-terminated PTHF was melted at 60 °C and dissolved in 35 mL of dichloromethane (DCM) by stirring in a round-bottom flask. Then, 2-isocyanatoethyl methacrylate was added to the PTHF solution in a 2:1 molar ratio, along with 0.06 mol% dibutyltin dilaurate as a catalyst. The reaction was maintained under vigorous stirring at room temperature (RT) for 48 h. Afterward, the excess DCM was removed using a rotary evaporator at 38 °C under a pressure of 400 mbar. The final product was then purified by precipitation in cold acetone followed by cold ether. The resulting methacrylated polytetrahydrofuran (mPTHF) was a viscous liquid at room temperature.

### 2.4. Synthesis of Cross-Linked Composite Electrolyte

In order to make crosslinked composite polymer film, precursor solutions were prepared by methacrylated-PTHF (mPTHF), PEGDA, LiTFSI salt and 2-hydroxy-2-methylpropiophenone (photoinitiator) and the composition variation is given in Table 1. First, mPTHF was mixed with PEGDA at a weight ratio of mPTHF:PEGDA = 9:1 (mPTHF/PEGDA) and LiTFSI salt was added after dissolving it in small amount of THF solvent (wt. ratio of LiTFSI:THF is 1:1) inside an argon-filled glovebox. The total amount of LiTFSI was calculated based on the molar ratio of the repeating units of PTHF to the LiTFSI salt, considering the ratio of oxygen atoms in PTHF to Li ions in LiTFSI, as well as the ratio of ethylene oxide (EO) groups in PEGDA to Li ions. Since the amount of PEGDA is negligible compared to that of PTHF, the O:Li ratio of 5:1 was kept for all prepared samples. Further, synthesized LATP particles were added to the mPTHF/PEGDA mixture with various weight ratios of 10, 15, 20 wt.%. After adding 1 wt.% of 2-hydroxy-2-methylpropiophenone (photoinitiator) the mixture was uniformly stirred at 300 rpm for 1 h using a magnetic stirrer. The solution was dropped between two glass slides, and UV-irradiated for 15 min to obtain the composite polymer electrolyte film.

### 2.5. Characterization and Measurements

The phase composition of LATP powder was analyzed using X-ray diffractometer (XRD) with a Rigaku SmartLab X-ray system (Rigaku, Tokyo, Japan). The system was equipped with a Cu X-ray tube and a D-Text detector, scanning over a 2θ range of 10–70° with a step size of 0.06°.

Structural analysis of the composite polymer electrolytes (CPEs) was carried out using Fourier-transform infrared spectroscopy (FTIR-ATR, Madison, WI, USA) in the frequency range of 400–4000 cm^−1^. Surface morphology was examined by field emission scanning electron microscopy (FESEM, JEOL JSM-7500F, Jena, Germany). Thermogravimetric analysis (TGA) was performed using a STA 6000 thermal analyzer (Waltham, MA, USA), heating the samples at a rate of 10 °C per minute under nitrogen flow, from 30 °C to 500 °C.

The ionic conductivity of the composite polymer electrolytes (SS|CPE|SS) was measured using electrochemical impedance spectroscopy (EIS) over a temperature range of 30–80 °C. A potentiostat/galvanostat (Metrohm AutoLab 204) was used to record measurements in the frequency range of 1 Hz to 1 MHz. The conductivity (σ) values were calculated using the equation:

$$\sigma =l/(R\pi r^{2})$$

where l is the thickness of the CPE, r is the radius of the CPE, and R is the bulk resistance.

The electrochemical stability window of the CPEs was determined using linear sweep voltammetry (LSV) at a sweep rate of 0.1 mV/s in the voltage range of 2–6 V at 80 °C, with SS|CPE|Li cell configuration.

Cyclic voltammetry (CV) was performed from 0 V to 5.0 V at a scan rate of 0.1 mV/s to evaluate the electrochemical performance of the Li|CPE|LFP cell configuration.

To prepare the cathode, LiFePO_4_ (LFP) powder was mixed with acetylene black (AB) and poly(tetrahydrofuran) (PTHF) in a mass ratio of 8:1:1. The mixture was dispersed in N-methyl-2-pyrrolidone (NMP) to create a homogeneous slurry, which was then applied to carbon-coated aluminum foil and dried in a vacuum oven.

Li/Li symmetric cells were assembled with the obtained composite polymer electrolyte (CPE) to evaluate the compatibility of the CPE with Li anode through Li plating/stripping cycling experiments. The tests were performed at a current density of 0.2 mA cm^−2^, with the temperature held at 80 °C.

The mechanical properties of the films were evaluated using a tensile testing machine (Materials Testing Machine Z010/TN2S, (Kennesaw, GA, USA)), with tests conducted at a tensile speed of 10 mm/min.

## 3. Results

PTHF-based films, characterized by low oxygen content and high chain flexibility, have the potential to improve ion transport in solid-state electrolytes [20,24,35]. In this study, a series of flexible composite solid polymer electrolyte (CPE) films were synthesized using methacrylated polytetrahydrofuran (m-PTHF) and Li_1.4_Al_0.4_Ti_1.6_(PO_4_)_3_ (LATP) via UV-induced crosslinking in the presence of PEGDA. The LATP content in the films was varied, while maintaining a constant Li salt concentration (O:Li = 5) and a fixed m-PTHF:PEGDA ratio of 9:1. Poly(ethylene glycol) diacrylate (PEGDA) oligomer was incorporated to enhance the mechanical stability, contributing to the formation of a robust crosslinked network with m-PTHF. The resulting CPE films were freestanding, flexible, and easy to handle. The detailed compositions of the CPEs are presented in Table 1.

**Figure 1:**
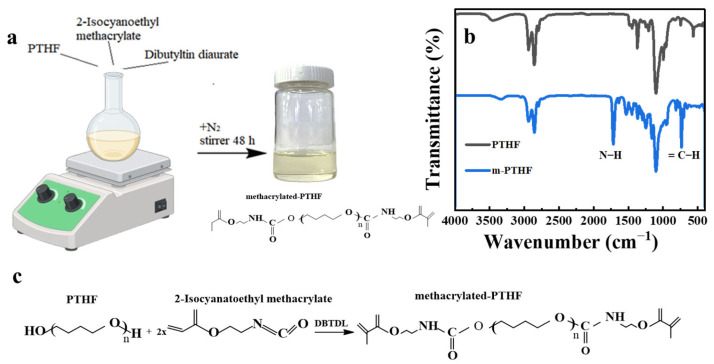
(**a**) Schematic illustration of the synthesis of methacrylated-PTHF; (**b**) FTIR Spectroscopy on polymer (α-methacrylated PTHF); (**c**) chemical reaction of the synthesis of methacrylated-PTHF.

Before preparing the CPE, the PTHF polymer was chemically modified by replacing its terminal hydroxyl groups (–OH) with methacrylate groups, as illustrated in the reaction scheme in Figure 1c. The successful functionalization of PTHF with dimethacrylate groups was confirmed by Fourier transform infrared (FTIR) spectroscopy (Figure 1b). The appearance of characteristic peaks at 1720 cm^−1^ and 730 cm^−1^ corresponds to the N–H and = C–H bending vibrations, respectively, indicating the successful introduction of methacrylate functionalities [20]. Additionally, the weakening of the –OH bond peaks around 3500 cm^−1^ suggests that most of the terminal groups have been replaced by methacrylate groups [36].

**Figure 2:**
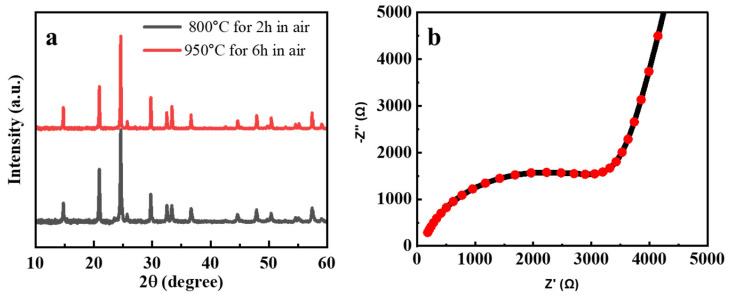
(**a**) XRD images of the prepared LATP in the form of powder and pellet; (**b**) impedance profile measured at room temperature for LATP pellet.

Ceramic LATP particles were synthesized using a straightforward and practical evaporation-induced self-assembly (EISA) method [37]. The X-ray diffraction (XRD) patterns of LATP powders synthesized at various temperatures are shown in Figure 2a, alongside the standard diffraction peaks of LiTi_2_(PO_4_)_3_ (JCPDS card No. 35-0754) for reference [38]. In all samples, the characteristic peaks of pure LATP with the NASICON-type structure are observed, indicating successful synthesis [13]. No significant differences in the crystalline phase are noted among the samples, suggesting that the temperature variations did not affect the phase purity. The lattice parameters, calculated from the XRD data, are a = 0.8471 nm and c = 2.0763 nm, which correspond to the R-3c space group.

The ionic conductivity of the LATP pellet measured by EIS and shown in Figure 2b. According to the results, its value reached 6.4 × 10^−5^ S·cm^−1^, which is comparable with the literature [33,39]. 

**Figure 3:**
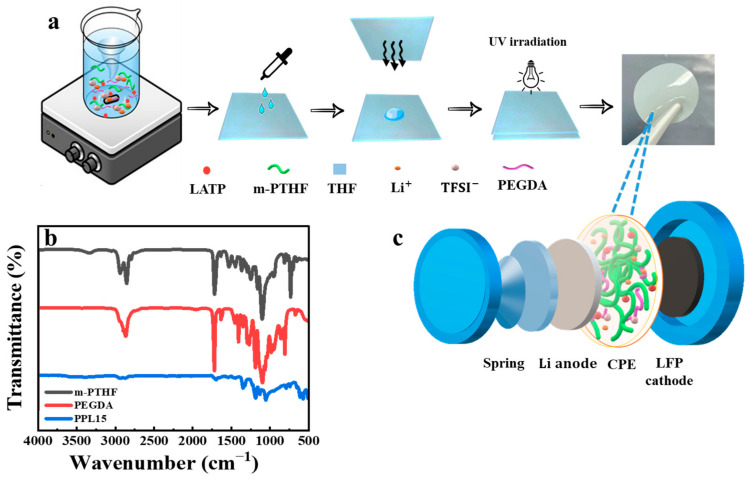
Scheme of CPE synthesis (**a**); FTIR Spectroscopy on polymer (α-methacrylated PTHF), PEGDA, CPE (**b**); structure of coin cell (**c**).

CPE films were synthesized through UV irradiation of a solution containing methacrylated polytetrahydrofuran (m-PTHF), polyethylene glycol diacrylate (PEGDA), LATP, and LiTFSI salt, as shown in the reaction scheme in Figure 3a. FTIR analysis of the UV-cured CPE films confirmed successful crosslinking, evidenced by the significant reduction in the characteristic acrylic bond peak at 730 cm^−1^ (Figure 3b) [40]. This reduction indicates that the majority of the carbon double bonds were consumed during the crosslinking process, resulting in a stable, interconnected polymer network. PEGDA acts as a crosslinker with repeating ethylene oxide units and reactive alkene end groups, enhancing the PTHF network by incorporating its solvating properties and providing mechanical and thermal stability. Moreover, the ester groups in PEGDA coordinate with lithium ions, promoting lithium salt dissociation and facilitating cation migration [41].

**Figure 4:**
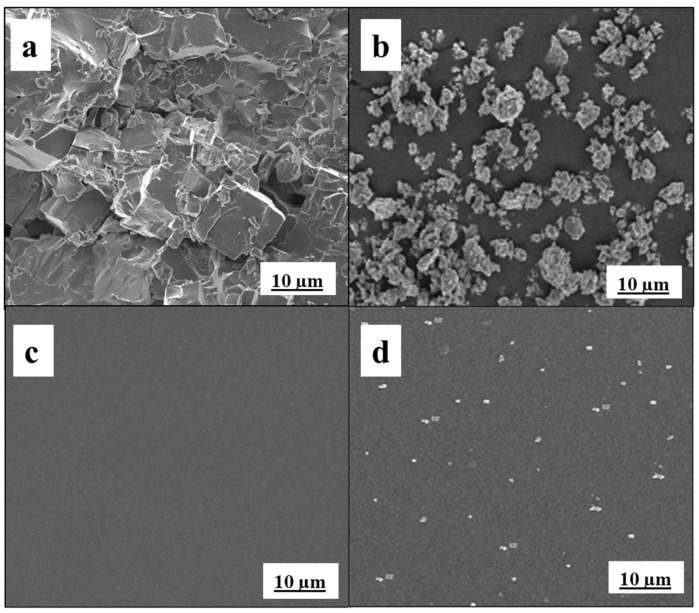
SEM images of (**a**) LATP pellet; (**b**) LATP powder; (**c**) PPL0; (**d**) PPL15.

The morphology of the m-PTHF and LATP-based films was analyzed using SEM, as shown in Figure 4. The microstructure of the LATP pellet and powders is shown in Figure 4a,b, respectively. The milling process standardizes the particle size distribution of the synthesized powder, leading to a uniform microstructure free of clustered pores or large grains. To prevent crack formation and maintain high ionic conductivity, it is crucial to avoid grain growth in the LATP microstructures, which can be influenced by the an-isotropic thermal expansion along the different crystallographic axes of the LATP structure. Notably, increasing the sintering temperatures from 800 to 950 °C for the milled powder does not cause any cracking [39]. Representative images of the films containing 0 wt% and 15 wt% LATP are given in Figure 4c,d, respectively. The results reveal a consistently smooth and uniform surface morphology across all polymer film samples without LATP (Figure 4c). Importantly, there is no evidence of agglomerated lithium salt particles, indicating that the lithium salt is effectively dissolved and uniformly integrated into the polymer structure. This uniform dispersion is essential for achieving consistent ionic conductivity and optimizing the overall performance of the composite solid polymer electrolyte [42]. Notably, the PPL15 film (containing 15 wt% LATP) demonstrates excellent compatibility with LATP particles evenly distributed throughout the polymer matrix. From the SEM images of the PPL15 composite polymer electrolyte containing 15% LATP particles, white crystals can be observed, corresponding to the LATP ceramic particles. This distribution is consistent with findings in other published works and demonstrates a homogeneous distribution of ceramic particles throughout the polymer matrix [43,44]. The thickness of the resulting CPE films ranged between 60 and 70 µm, ensuring uniformity across the samples.

**Figure 5:**
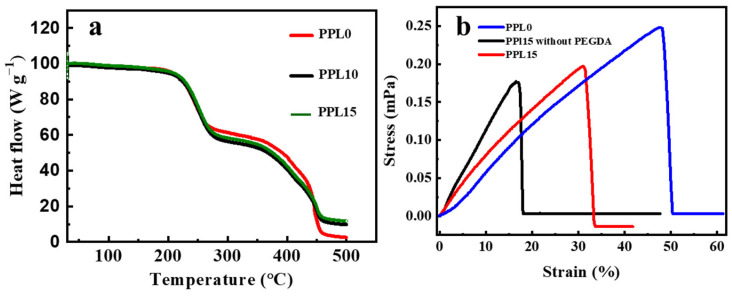
TGA of PPL CPEs (**a**); stress-strain curves of PPL CPEs (**b**).

To assess the stability of the LATP-containing CPE based on m-PTHF for lithium-ion battery applications, its thermal stability was analyzed using thermogravimetric analysis (TGA). The TGA trace for the m-PTHF/LATP composite, presented in Figure 5a, indicates an initial decomposition temperature of 250 °C, which is attributed to the breakdown of tetramethylene oxide units in m-PTHF [24]. A second decomposition phase, occurring at 350 °C, is directly attributed to the networked structure formed between a-PTHF and PEGDA molecules during the crosslinking process [40]. According to the TGA results, the thermal stability of the crosslinked PPL films exceeds the safety threshold typically required for lithium-ion battery operation.

Figure 5b presents the stress-strain curves for m-PTHF/LATP CPEs containing 0 wt% and 15 wt% LATP by mass, as well as m-PTHF/LATP CPEs without a crosslinking oligomer to assess the impact of PEGDA and LATP. The PPL15 film without PEGDA demonstrated a tensile strength of 0.17 MPa and a breaking strain of 18% which is about 2.5 lower than those of the PPL15 film crosslinked with PEGDA. The increase in elongation observed with the addition of oligomer is likely due to the higher average molecular weight of the polymer and the enhanced chain flexibility provided by the ethoxy groups [1,45]. However, both mechanical strength and elongation results are lower in comparison with the films without ceramics (PPL0). The reason for this may be poorly shaped or oversized nanoparticles that can act as flaws or stress concentrators, lowering the film’s strength and increasing its brittleness. Overall, the mechanical strength of PPL15 CPE is sufficient for use as a freestanding solid electrolyte in lithium-ion batteries [46]. Nevertheless, despite the reduction in fracture strain, it retained its flexibility and good mechanical strength, as shown in Figure 3a.

**Figure 6:**
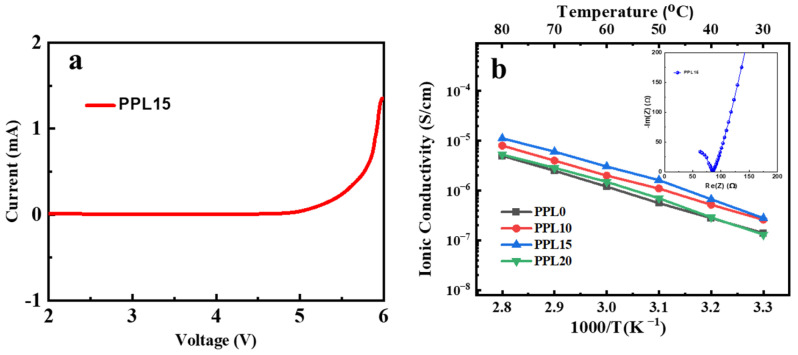
LSV curve of PPL15 CPE (**a**); Arrhenius plots for PPL CPEs with different LATP content (**b**).

The ability of membranes to remain stable over a wide potential range is crucial for their application in lithium-ion battery systems. To evaluate the stability of the CPE against reduction and oxidation processes, linear sweep voltammetry (LSV) measurements were performed. LSV measurements showed that PPL15 CPE exhibited no significant oxidation and reduction peaks up to 5.2 V (Figure 6a). The CPE PPL15 demonstrated excellent electrochemical stability, with a similar profile suitable for high-voltage lithium-ion batteries.

Battery cells with a stainless steel|SPE|stainless steel (SS|SPE|SS) configuration were assembled using the PPL CPEs, and electrochemical impedance spectroscopy (EIS) measurements were performed.

The temperature-dependent ionic conductivity of PPL CPEs with varying LATP content is shown in Figure 6b. Based on the EIS results, the ionic conductivity of PPL CPEs increases with both rising temperature and higher ceramic particle content, reaching a maximum value of 1.57 × 10^−5^ S·cm^−1^ for PPL15, which contains 15 wt% LATP. The overall homogeneous distribution of ceramic particles promotes optimal conditions for enhanced ion migration [43]. LATP particles, with their intrinsic high ionic conductivity due to Al³⁺ ions, create additional pathways for Li⁺ migration. In a composite polymer electrolyte, LATP establishes conductive ion channels, forming a percolative network that reduces pathway tortuosity and facilitates efficient Li⁺ transport within the polymer matrix. The sharp decline in ionic conductivity for the sample with 20 wt% LATP can be attributed to the agglomeration of ceramic particles within the CPE matrix [47]. This could potentially lead to the collapse of the ceramic percolation network and the disruption of the ion-conducting pathways in the ceramic material. Consequently, this would also disrupt the continuity of lithium-ion transport pathways within the polymer matrix, caused by the higher concentration of ceramic particles. The inset in Figure 6b presents a representative impedance plot for the PPL15 CPE at 80 °C. The ionic conductivity of the PPL0 system, without LATP particles, was also tested, reaching 5·10^−6^ S·cm^−1^ at 80 °C. The inclusion of LATP directly contributes to increasing the overall ionic conductivity by introducing highly conductive ceramic pathways [45]. Also, it makes lithium ions move more easily by increased polymer chain mobility. The LATP particles increase the surface area within the polymer matrix, providing more interfaces where ion hopping or ion exchange can occur. These interfaces between LATP and the polymer phase act as regions where ions can transfer quickly from one medium to another. This mechanism is complex and influenced by various factors, including ionic conductivity, lithium-ion transference number (tLi^+^), and the polymer microstructure [48].

The CV-profile indicated in Figure S1 of the Supplementary Materials shows the typical oxidation and reduction peaks for LFP cathode. The reduction of the oxidation peak at 0.1 mA may be attributed to kinetic and mass transport limitations, as well as potential side reactions that could diminish the effective current response. Two characteristic peaks of the CV curve of the cell with LFP cathode, CPE and Li anode could be easily seen. The oxidation and reduction peaks were detected at potential around 3.28 V and 3.95 V, respectively [49].

The slight shift of the peaks compared to the literature (3.3 V, 3.6 V) indicates higher polarization in the battery. Additionally, structural changes in the electrode material or limitations in the ionic conductivity of the electrolyte also contribute to this observation [50,51]. The stripping/plating test was conducted at 0.2 mA·cm^−2^ and 80 °C using a Li/PPL15/Li cell to evaluate the electrolyte’s compatibility with the lithium anode. The cell demonstrated a low polarization potential of approximately 30 mV over 100 h (Figure S1b). This low polarization potential can be attributed to the efficient lithium ion transport within the composite polymer electrolyte, which facilitates rapid intercalation and deintercalation processes [51]. The obtained results suggest that the fabricated PTHF-based crosslinked composite polymer electrolyte, incorporating LATP and lithium salt, is a promising system with high ionic conductivity, improved mechanical strength, and maintained flexibility. These properties make it a competitive candidate for application in lithium-ion batteries, comparable to other advanced flexible composite polymer electrolyte systems.

## 4. Conclusions

In this study, we successfully developed a novel composite polymer electrolyte (CPE) by integrating crosslinked polytetrahydrofuran (PTHF) with polyethyleneglycol diacrylate (PEGDA) and lithium aluminum titanium phosphate (LATP) particles. The resulting CPEs demonstrated significant improvements in mechanical strength, ionic conductivity, and electrochemical stability, particularly in formulations containing 15% LATP. The optimal ionic conductivity observed, coupled with the films’ robust thermal stability, indicates their potential applicability in high-performance lithium-ion batteries (LIBs). The ionic conductivity value of PPL15 CPE was 1.57·10^−5^ S·cm^−1^ at 80 °C. Additionally, the successful mitigation of oxidation up to 5.2 V underscores the electrochemical resilience of the developed CPEs, making them suitable for high-voltage applications. These findings highlight the importance of optimizing both the composition and structure of composite electrolytes to achieve superior performance in solid-state battery systems.

## Figures and Tables

**Table 1:** Composition of CPEs based on m-PTHF.

No.	Sample Name	mPTHF:PEGDA = 9:1 (wt. %)	LATP (wt %).	O:Li
1	PPL0	100	0	5
2	PPL10	90	10	5
3	PPL15	85	15	5
4	PPL20	80	20	5

## Data Availability

All data generated or analysed during this study are included in this published article.

## Supplementary Materials

The following supporting information can be downloaded at: https://www.mdpi.com/article/10.3390/polym16223176/s1, Figure S1. (a) The CV-curves of LFP/PPL15/Li cell, (b) Profile of galvanostatic cycling potential for Li–Li symmetrical cell with PPL15 CPE cycled at 0.2 mA·cm^−2^.
