# Supplementary material for: PTHF/LATP Composite Polymer Electrolyte for Solid State Batteries
Source: Polymers (Basel). 2024 Nov 14;16(22):3176. doi: 10.3390/polym16223176 (PMC11598488; doi:10.3390/polym16223176)
Supplement: Supplementary file 1 [file polymers-16-03176-s001.zip › polymers-3298781-supplementary.pdf]

PTHF/LATP Composite Polymer Electrolyte for Solid State Batteries

Elmira Nurgaziyeva[1], Gulnur Turlybay[1], Aigul Tugelbayeva[1], Almagul Mentbayeva[2], Sandugash Kalybekkyzy[3*]

[1] *National Laboratory Astana, Nazarbayev University, Astana, 010000, Kazakhstan*

[2] *Department of Chemical and Materials Engineering, School of Engineering and Digital Sciences, Nazarbayev University, Astana, 010000, Kazakhstan*

[3]*Department of Chemistry, School of Sciences and Humanities, Nazarbayev University, 010000, Astana, Kazakhstan*

***sandugash.kalybekkyzy@nu.edu.kz**

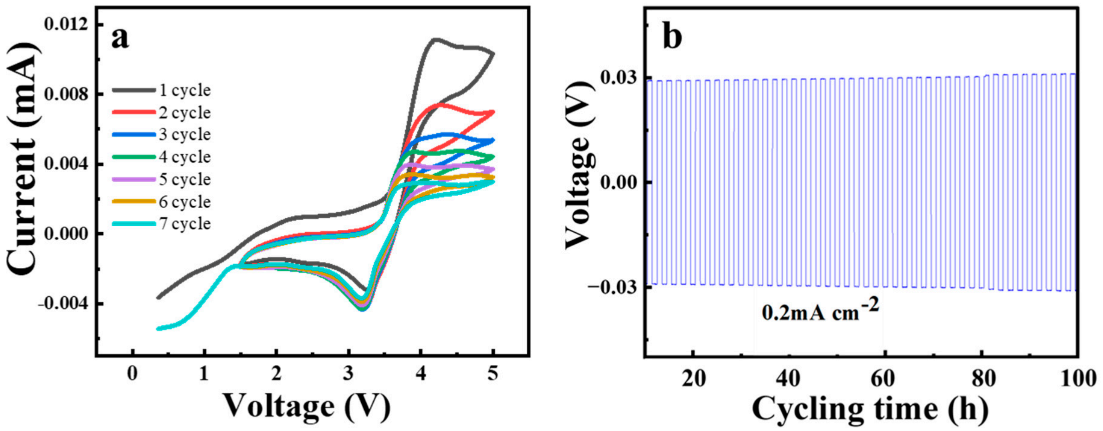

Figure S1. a) The CV-cirves of LFP/PPL15/Li cell, b) Profile of galvanostatic cycling potential for Li–Li symmetrical cell with PPL15 CPE cycled at 0.2 mA·cm^{-2}
